# Metal on Metal Bearing in Total Hip Arthroplasty and Its Impact on Synovial Cell Count

**DOI:** 10.3390/jcm9103349

**Published:** 2020-10-18

**Authors:** Henrik C. Bäcker, Chia H. Wu, David Krüger, Clemens Gwinner, Carsten Perka, Sebastian Hardt

**Affiliations:** 1 Department of Orthopaedic Surgery and Traumatology, Charité Berlin, University Hospital, Charitéplatz 1, 10117 Berlin, Germany; clemens.gwinner@charite.de (C.G.); Carsten.perka@charite.de (C.P.); Sebastian.hardt@charite.de (S.H.); 2 Department of Orthopedics & Sports Medicine, Baylor College of Medicine Medical Center, Houston, TX 77030, USA; wu.chia.h@gmail.com; 3 Department of Orthopaedics, Herzogin Elisabeth Hospital, 38124 Braunschweig, Germany; d.krueger@heh-bs.de

**Keywords:** metal on metal, total hip arthroplasty, aspiration, infection, cell count, marker

## Abstract

Introduction: The effect of different bearings on synovial white blood cell (WBC) count and polymorphonuclear percentage (PMN%) in aspirations remains unclear. Therefore, this study investigates the impact of aseptic Metal-on-Metal (MoM) bearing on synovial fluid. Methods: We searched our arthroplasty registry for aseptic painful THAs with MoM bearings between 2011 and 2018. Then, a case-matched control group was selected with septic and aseptic Total Hip Arthroplasty (THA) with ceramic on a polyethylene (PE) bearing. The matching criteria consisted of gender, age +/−10 years, and time of aspiration (+/−2years). Periprosthetic Joint Infection (PJI) was defined according to the Infectious Diseases Society of America (IDSA), and Musculoskeletal Infection Society (MSIS) using bacterial cultures, sonication and histology. Results: In total, 19 patients who underwent hip aspiration with MoM bearing were identified. Five patients had to be excluded due to insufficient synovial fluid obtained (*n* = 2) or bacterial growth after sonication that was initially negative with the standard microbiological cultures (*n* = 3). As such, 14 were included. These patients were matched with 14 aseptic and 14 septic THAs with ceramic on a PE bearing, which constituted the control group. The mean serum chrome level was 20.0 ± 15.5 nmol/L and cobalt level 18.4 ± 22.1 nmol/L. The synovial WBC and PMN% varied significantly between MoM bearing group and the aseptic THA ceramic PE group (both *p* < 0.001), as well as the septic THA group (WBC *p* = 0.016, PMN% *p* < 0.001). Furthermore, the septic THA group had significantly higher CRP values than the aseptic MoM group (*p* = 0.016). Conclusion: MoM bearing shows significantly higher synovial WBC and PMN% when compared to aseptic THA with ceramic on PE bearing above the MSIS cut-off. This is an important consideration when diagnosing periprosthetic joint infection using the MSIS guidelines.

## 1. Introduction

In total hip arthroplasty (THA), different bearings are being used, including metal-on-metal (MoM) and ceramic on polyethylene (PE) bearings. In 1988, Weber et al. reintroduced the second generation of MoM bearings to minimize the PE wear complications, which resulted in periprosthetic osteolysis [1]. Because of increased failure rate and adverse local tissue reaction related to the metal wear, only approximately 35% of all THA replacements in the US consisted of MoM bearing in 2008 [2,3,4,5,6]. Although these bearings are not used as often anymore, the white blood cell count is highly important in the diagnosis of PJI as well as metal toxicity, as many of these patients need revision due to corrosion, osteolysis and pseudotumor [7].

To exclude other causes of loosening, such as periprosthetic infection (PJI), most guidelines recommend plain films to assess for osteolysis and metal wear, in addition to serum-based infection parameters and joint aspirates, before proceeding with surgery [8]. Therefore, synovial fluid white blood cell count, monomorphonuclear (MMN) and polymorphonuclear cells (PMN), as well as the PMN%, are needed for decision making [9]. In the literature, there is an ongoing debate on the cut-offs for synovial WBC to distinguish between aseptic and septic THA. Currently, these range, for native, aseptic THAs, from 1800 to 3000 lc/µL, as recommended by the Musculoskeletal Infection Society (MSIS), the Infectious Diseases Society of America (IDSA) and the New Diagnostic Criteria for Periprosthetic Joint Infection [10,11,12]. 

However, no study to date has investigated the influence of different bearings—metal on metal versus polyethylene on ceramic—on synovial inflammatory markers. This study aims to assess the impact of bearing choice in THAs on synovial and serum inflammatory markers, in both aseptic and septic states.

## 2. Methods

A retrospective chart and radiographic review were performed between November 2015 and 2019. All consecutive patients with aseptic, painful metal-on-metal bearing THAs who underwent hip aspiration and revision surgery due to aseptic loosening were included. The inclusion criteria consisted of patients who are 18 years old or older, serum inflammatory markers including synovial fluid differentiation, synovial blood count (WBC), polymorphonuclear distribution in percentage (PMN%), as well as erythrocyte count and postoperative histology, microbiology cultures and sonication results. Histology was classified according to Morawietz L et al. [3]. For microbiology cultures, probes were incubated for 14 days to diagnose even slow-growing bacteria species. In cases of bloody aspiration, synovial WBC were corrected. Therefore, the following formula was applied: WBC adjusted = WBC observed − (WBCblood × RBC fluid/RBC blood) [13]. In addition, serum chrome and cobalt levels were noted [13].

All cases were matched to patients with primary aseptic THAs as well as septic total hip arthroplasties. Matching criteria included gender, age plus or minus 10 years, year of surgery plus or minus 2 years. In both aseptic and septic cases, the control group used polyethylene inlays with ceramic femoral heads. 

Likewise, for the metal-on-metal group, infection was diagnosed according to the MSIS 18 and IDSA criteria as illustrated in Table 1 [10]. An extended culture protocol of 14 days was performed. If microbiology was positive, we noted the interpretation of the infectious disease specialist. Patients who underwent total hip arthroplasty within one year, or had a history of previous periprosthetic joint infection (PJI), previous use of revision implants, and any other structural deformities such as inlay wear or implant failure were excluded. Furthermore, we noted the serum inflammatory markers including the WBC count and c-reactive protein (CRP).

For statistical analysis, Origin Pro 8.0, Microsoft Excel and IBM SPSS Statistics 25 were used. A one-way *t*-test, and post hoc least significant difference test (LSD) for descriptive statistics were applied to calculate the aspirate differentiation, cell count, PMN% and monomorphic nuclear cells (percentage) for categorical variables. The mean and standard error of the mean (SE) were used for normally distributed continuous variables. 

## 3. Results

A total of 19 patients with metal-on-metal bearing were identified during the studied time period. Two patients (*n* = 2/19) were excluded because not enough synovial fluid (less than 2 mL) was obtained. Further, three patients showed bacterial growth after sonication that was initially negative with the standard microbiological cultures. This left 14 patients for inclusion, which were matched with 14 aseptic and 14 septic THAs with ceramic on PE bearing, as previously described. Gender was equally distributed, with seven patients in each (50.0%; *n* = 7/14). The mean age at time of aspiration was 64.6 ± 12.2 years old for the MoM group, 66.0 ± 10.8 years old for the aseptic group, and 67.5 ± 21 years old for the septic THAs (*p* = 0.752). 

In the MoM group, the mean WBC and CRP in serum were 9.1 ± 2.6 G/L (reference range 3.9–10.2 G/nL) and 8.8 ± 14.2 mg/dL (reference range <5 mg/dL), respectively. The serum chrome level was 20.0 ± 15.5 nmol/L (reference range 1–8 nmol/L). The cobalt level was 18.4 ± 22.1 nmol/L (reference range <12 mmol/L). In the aspirate, the synovial WBC was 3198 ± 2452 lc/µL, including 857 ± 787 lc/µL polymorphonuclear cells. PMN% was reported to be 31.8 ± 20.9%, eosinophil cells at 24 ± 35 lc/µL, and erythrocytes in 126 ± 228 lc/µL. For histology, in nine patients, foreign body particles including macrophages and multinucleated giant cells were detected (type I; *n* = 9/14, 64.3%), and in three cases, a periprosthetic membrane of indeterminate type formed by connective tissue (type IV; *n* = 3/14, 21.4%) was observed. In three patients, histology revealed low-grade infection (*n* = 3/14, 21.4%) but no specific organism was isolated in microbiological cultures. Therefore, after interdisciplinary discussion with the infectious disease specialist, we decided to treat these patients as aseptic according to the MSIS and IDSA criteria. 

For the aseptic group, mean serum WBC was 8.5 ± 3.8 G/L and CRP 29.6 ± 50.7 mg/dL. In the aspirate, synovial WBC was 711 ± 391 lc/µL with 333 ± 302 lc/µL polymorphonuclear cells (PMN% was 45.0 ± 21.0%). The eosinophil cell count was 21 ± 29 lc/µL with 311 ± 302 lc/µL erythrocytes. In all cases, histology as well as microbiology revealed no pathological findings.

The infected THA group showed higher serum WBC and CRP at 6.7 ± 1.8 G/L and 38.3 ± 38.9 mg/dL, respectively. In joint aspirate, the synovial WBC was 14,946 ± 16,380 lc/µL with 13,361 ± 15,339 lc/µL of polymorphonuclear cells. The PMN% was reported at 77.6 ± 23.0%. The eosinophil and erythrocyte count was slightly higher compared to the other groups at 406 ± 781 and 421 ± 676 lc/µL, respectively. According to MSIS as well as IDSA criteria, all patients met the criteria for PJI. In 10 of 14 cases, microbiology cultures revealed bacterial growth. In most cases, *staphylococcus epidermidis* was identified (*n* = 4/10), followed by one case each of *enterococcus faecium*, *streptococcus sanguinis*, *staphylococcus aureus*, *bacillus cereus*, *pseudomonas acnes*, and *paenibacillus species* (*n* = 6/10). Additionally, ten cases had a histology that revealed either low-grade (type III, *n* = 5/8, 70.0%) or high-grade infection (type II, *n* = 3/8, 37.5%). All findings are listed in Table 2.

Between the different groups, a couple of significant findings were identified. Serum CRP was significantly higher for the septic compared to the MoM group (*p* = 0.048). However, no statistical significances were found for the serum WBC. For the aspirate, synovial WBC varied significantly between the individual groups. It was lowest for the aseptic group, followed by the metal-on-metal group (aseptic vs. MoM, *p* = 0.001). For the infected THAs, multiple comparisons were significant (MoM vs. septic *p* = 0.017; aseptic vs. septic *p* = 0.004) with a post hoc Least Significant Difference (LSD) *p* value of 0.001. Similar findings were observed for the polymorphonuclear cells (aseptic vs. MoM *p* = 0.034; MoM vs. septic *p* = 0.007; aseptic vs. septic *p* = 0.005) at a post hoc LSD *p* value of <0.001. Also, PMN% was significantly higher for the septic group, followed by the aseptic hip and MoM groups (aseptic vs. MoM *p* = 0.117; MoM vs. septic *p* < 0.001; aseptic vs. septic *p* < 0.001). No significant differences were found between aseptic and metal on metal bearing THAs (*p* = 0.48).

## 4. Discussion

Our study shows that the bearing of total hip arthroplasties has a significant impact on the synovial white blood cell count (*p* = 0.001) as well as polymorphonuclear cell distribution (*p* ≤ 0.001). The mean WBC in aseptic MoM according to the MSIS and IDSA criteria was 3198 ± 2452 lc/µL, which exceeds even the threshold recommended by the MSIS [11].

Synovial fluid is produced by synovial cells that can cause interstitial fluid shifts through a pressure gradient. This can be influenced by particles from metal wear [15,16]. Waterson et al. described an increase in major complications when using metal bearings, such as deep infections in up to 18.2% of patients [17]. Additionally, other studies reported that, due to an adverse local tissue reaction in metal on polyethylene bearing, periprosthetic infection can be under-diagnosed in the context of trunnion corrosion [18]. On the other hand, Della Valle et al. described that the presence of adverse local tissue reaction can also mimic periprosthetic joint infection as well as aseptic loosening [19]. 

It is known that metal particles have antibacterial properties. Research has shown that cobalt can reduce bacterial count after 8 and 24 h of exposure, especially for *Escherica coli* [20]. However, the impact of chrome particles on bacteria has not been shown to our knowledge. Therefore, Kwon et al. suggested an optimal cut-off value for synovial WBC with 2144 with 93% sensitivity and 84% specificity, although no differences in metal ion levels between infected and non-infected groups were found [21]. A variety of different cut-offs have been described in the literature, ranging from 1800 lc/µL to as high as 3000 lc/µL with PMN distribution between 60% and 89%, but there is no consensus [8,10,14,22]. This may explain why the MSIS considers the synovial aspiration only as a minor criteria [11]. In the newly developed MSIS diagnostic criteria for PJI, the threshold for synovial WBC count is 3000 lc/µL [8,11].

Within the 14 patients included in our study, mean chrome level (20.0 ± 15.5 nmol/L) and cobalt level (18.4 ± 22.1 nmol/L) was elevated. No correlation between serum leucocytes was found. However, CRP correlated most strongly with the septic group, followed by aseptic THAs and MoM group (*p* = 0.121). Significant differences were found for synovial white blood cells and PMN% for MOMs at 3198 ± 2452 lc/µL and 31.8 ± 20.9%. Aseptic THAs is reported at 711 ± 391 lc/µL and 45.0 ± 21.0%, respectively. Finally, septic group is reported at 14,946 ± 16,380 lc/µL and 77.6 ± 23.0% (*p* < 0.001), respectively.

However, the PMN% was lower for the MoM group when compared to the aseptic THAs group (31.8 ± 20.9% vs. 45.0 ± 21.0%). It has to be mentioned that the mean synovial WBC in the MoM group outranged the MSIS threshold. All microbiology cultures and sonication remained sterile. In three patients, histology showed an intermediate type of corrosion with suspicion of a low-grade infection—type three, according to Morawietz L et al. These cases were not considered to be PJI and were treated as contamination [23].

This study is limited by its retrospective design, describing only a small cohort of 14 patients in each group. The mean cobalt and chrome levels were elevated and well above the serum reference, in part because of metal particles from wear. This can lead to a culture negative infection which typically ranges between 0% and 42.1% [24]. Therefore, all patients even with only bacterial growth in sonication were excluded. Within our cohort, three patients had histology results that showed signs of low-grade infection, despite all cultures and sonication remaining negative. According to the literature, positive histology is equally likely to be observed in osteomyelitis with low sensitivity and specificity [25]. Therefore, after interdisciplinary discussion with infectious disease specialists, these patients were treated as aseptic MoM according to MSIS and IDSA criteria. In the aseptic group, slight differences may occur between aseptic loosening and PE wear. This is likely because of particle debris, although we did not identify any in our cohort [26]. 

## 5. Conclusions

Synovial white blood cell count in metal on metal bearing is critical in order to agree on how to interpret findings in order to decide on the subsequent treatment. This study shows that the bearing has a significant impact on the synovial WBC count above the MSIS cut-off. The diagnosis of periprosthetic joint infection requires multiple measures like serum-based or synovial-based inflammatory markers. Furthermore, sonication, microbiological cultures and histology after THA removal also play a role. Therefore, other diagnostic tools should be considered to exclude infection. 

## Figures and Tables

**Table 1:** MSIS—Musculoskeletal Infection Society and IDSA—Infectious Diseases Society of America classification for periprosthetic joint infection; CI—chronic infection, AI—acute infection, CRP—c-reactive protein, lc—leucocytes adopted from [14].

	MSIS (≥1 of the 2 Major Criteria or ≥3 of 5 Minor Criteria	IDSA (≥1 of the Following 4 Criteria)
Major criteria	2 positive periprosthetic cultures	Purulence without other aetiology surrounding the prosthesis
	Sinus tract communicating with the prosthesis	Sinus tract communicating with the prosthesis
		Acute inflammation seen on histopathological examination of the periprosthetic tissue
Minor criteria	CRP>10 mg/L in CI, >100 mg/L AI and ESR >30 mm/h	≥2 intraop. Cultures
	Synovial fluid WBC >3000 lc/µL in CI or >10,000 lc/µL in AI	Combination of preop. aspiration and intraop. cultures yielding an indistinguishable organism
	Positive leucocyte esterase strip test (++ or +++)	
	Synovial fluid percentage of granulocytes >80% in CI or >90% in AI	
	Single positive culture	
	Positive histological analysis of periprosthetic tissue	

**Table 2:** Demographics, blood diagnostics and synovial fluid distribution.

	Age (Years)	Lc (G/L)	CRP (mg/dL)	WBC (lc/µL)	MMN (lc/µL)	PMN (lc/µL)	PMN% (%)	Eosinophils (lc/µL)	Erythrocytes (lc/µL)
MoM	64.6 ± 12.2	9.1 ± 2.6	8.9 ± 14.2	3198 ± 2452	1748 ± 1738	857 ± 787	31.8 ± 20.9	24 ± 35	126 ± 228
Aseptic	66.0 ± 10.8	8.5 ± 3.8	29.6 ± 50.7	711 ± 391	400 ± 300	333 ± 302	45.0 ± 21.0	21 ± 29	311 ± 302
Septic	67.5 ± 10.4	6.7 ± 1.8	38.3 ± 38.9	14,946 ± 16,380	1575 ± 1235	13,361 ± 15,339	77.6 ± 23.0	406 ± 781	421 ± 676
Signif.	0.771	0.107	0.121	0.001	0.022	<0.001	<0.001	0.062	0.245
